# Absorbable suture can be effectively and safely used to close the mesenteric defect in a gastric bypass Sprague-Dawley rat model

**DOI:** 10.1186/s12893-019-0671-9

**Published:** 2020-01-10

**Authors:** Libin Yao, Ponnie Robertlee Dolo, Yong Shao, Chao Li, Jason Widjaja, Jian Hong, Xiaocheng Zhu

**Affiliations:** 1 Department of General Surgery, The Affiliated Hospital of Xuzhou Medical University, Xuzhou, 221006 Jiangsu People’s Republic of China; 2 Institute of Digestive Diseases, Xuzhou Medical University, Xuzhou, 221006 Jiangsu People’s Republic of China

**Keywords:** Mesenteric defect, peterson’s space, Non-absorbable suture, Absorbable suture

## Abstract

**Background:**

To observe if closing the mesenteric defect with absorbable sutures creates a safe adhesion compared to non-absorbable suture after Roux-en-Y gastric bypass.

**Methods:**

Rats were randomly assigned to 5 experimental groups according to the different suture materials used in closing the mesenteric defects (Peterson’s space) after Roux-en-Y gastric bypass. Group A (control group), Group B (non-absorbable suture, Prolene suture), Group C (biological glue), Group D (non-absorbable suture, polyester suture) and Group E (absorbable suture). All rats were followed up for 8 weeks postoperatively and underwent laparotomy to observe the degree of adhesion and closure of the mesenteric defect.

**Results:**

No significant difference was found in the decrease in food intake and body weight among all groups. No internal hernia (IH) occurred in any group. The mesenteric defects of Group A remained completely visible without any closure or adhesion. Multiple gaps were found between the Prolene suture and the mesentery along the suture line in Group B. The mesenteric defects of Group C were complete closed with multiple adhesions of the small intestine and the greater omentum. The mesenteric defects in both Group D and Group E closed completely. The average adhesion scores in Group A and Group B were 0 and 0.33 ± 0.52 respectively. The average adhesion score in group C (3.83 ± 0.41) was higher than the other groups (*p*<0.05). The average adhesion scores in Group D and E were similar (3.17 ± 0.41 and 3.00 ± 0.00 respectively).

**Conclusion:**

Absorbable suture created a safe adhesion score between the mesentery which was not inferior to non-absorbable sutures.

## Background

Internal hernia (IH), which leads to small bowel obstruction (SBO) is an important long-term clinical complication after Roux-en-Y Gastric Bypass (RYGB) surgery. It can occur many years after the primary surgery or throughout life with serious consequences; often requiring extensive intestinal resection. Incidence of IH range from 1 to 5% [1–3].

After an antecolic RYGB surgery, IH typically occurs at the Peterson Space (space between Roux limb’s mesentery and transverse mesocolon) mesenteric defect, and the Brolin space (space between the mesentery at the jejuno-jejunostomy). It is generally accepted that closure of the mesenteric defect reduces the risk of IH thereby reducing the occurrence of SBO due to IH [4–9]. However, a study by Stenber g et al. [10], suggested that closure of the mesenteric defects could potentially increase the risk of early small bowel obstruction caused by kinking of the jejunojejunostomy. Even so, most studies still suggests that routine closure of mesenteric defect could reduce the risk of IH.

But even with the generally accepted practice of closing of mesenteric defects, there is still no consensus as to what sutures material should be used for closing the mesenteric defect. Most surgeons prefer to close mesenteric defect using non-absorbable sutures which is even recommended by the ASMBS (American Society for Metabolic and Bariatric Surgery) [11]. The reason for not using absorbable sutures is mainly due to concerns that the mesenteric defect may reopened once the absorbable sutures are absorbed after surgery, potentially leading to the occurrence of intra-abdominal hernia.

With the development of surgical sutures, absorbable sutures are being used extensively in clinical practice due to their good tensile strength, tissue compatibility and absorbability [12, 13]. But it remains unclear, whether closing the mesenteric defect with absorbable sutures creates a safe adhesion compared to non-absorbable suture. This remains unknown in current practice.

In this study, we established a Rat model of Roux-en-Y gastric bypass surgery and used different sutures to close the mesenteric defect (Peterson’s space) in order to explore whether closing the mesenteric defect with absorbable suture creates a safe adhesion compared to non-absorbable suture.

## Methods

### Study design

This study was approved by the ethics committee of Xuzhou Medical University Research Animal Centre. All applicable institutional and national guidelines of the People’s Republic of China for the care and use of animals were followed [14]**.**

Thirty male Sprague-Dawley obese rat (weight 350-380 g) purchased from Xuzhou Medical University Research Animal Center were randomly divided into 5 groups (*N* = 6). Rats in all groups underwent Roux-en-Y gastric bypass. After the gastrointestinal reconstruction during the RYGB, the Peterson’s space mesenteric defects were managed using different methods and suture materials namely: (A) No intervention/closure, control group; (B) Closure using non-absorbable prolene suture group (Ethicon Prolene Polypropylene Suture 4–0); (C) Closure using biological glue group (Compon/kangpaite biological adhesive, Beijing); (D) Closure using non-absorbable polyester suture group (Ethicon Polyester suture 4–0); (E) Closure using absorbable suture group (Covidien Polysorb Braided Absorbable Suture 4–0). Performing the gastric bypass in obese rat also enabled us to assess changes in the mesentery after weight loss and the effect on the adhesion using the different sutures.

All rats were followed for 8 weeks after surgery. Rodents in captivity are known to live for up to 3 years; making every day in the life of a rat equivalent to 35 human days [15]. Therefore 8 weeks in rodent will equate to approximately 5.4 human years. We therefore believe that 8 weeks was a reasonable time to assess the changes in the mesentery defect following the closure.

Following the end of the study, the rats were safely and ethically euthanized through overdose of isoflurane (exposure to 3 ml isoflurane using cotton ball as absorbant for 3–5 min using open-drop method). Isoflurane exposure was continued for approximately one-minute after breathing stops.

### Preoperative care

Rats were housed individually under constant ambient temperature and humidity on a 12 h day/night cycle. Rats were allowed free access to normal chow. Food intake was measure on a daily basis and average weekly. Each animal was housed in a shoe-box cage and given a fixed amount of food daily. To get the amount of food intake every day, we simply subtracted the amount of food leftover after 24 h from the fixed amount of food given in the beginning. The difference was recorded as the amount of food intake. The study was approved by the ethics committee of Xuzhou Medical University Research Animal Center.

### Surgical procedure

**Fig. 1:**
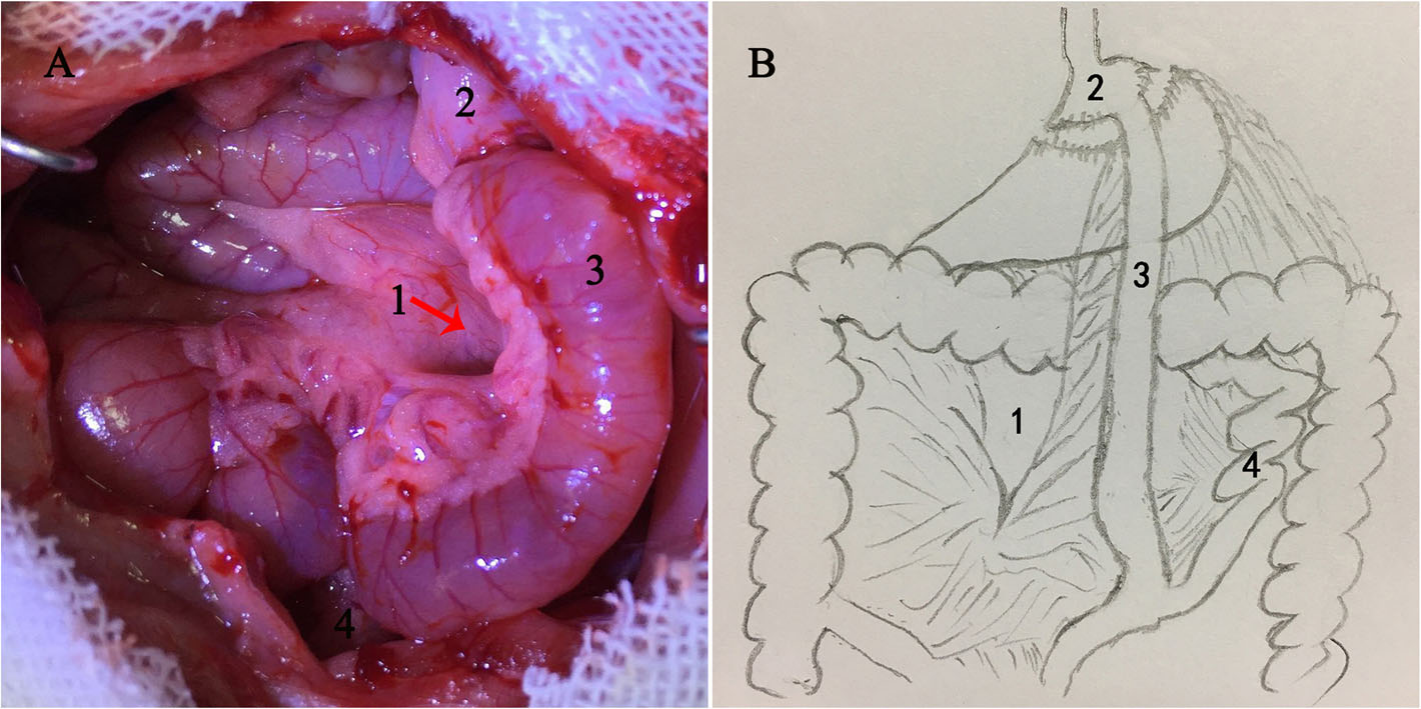
Illustrations of Peterson’s space formed in Roux-en-Y gastric bypass (RYGB). **a** RYGB surgery of rats (**b**) Sketch of RYGB surgery. 1. Peterson’s space 2. Small stomach pouch 3. Roux-limb 4. Biliopancreatic limb

After overnight fast, at approximately nine a.m. rats were sedated with 5% Chloral Hydrate (0.5 ml/100 g) through intraperitoneal injection. Under strict sterile condition, the rat was placed on the operating table and the incision site (mid-abdomen) cleaned with 5% povidone iodine without hair removal. An approximately 5 cm mid-line incision was made. RYGB was performed in similar fashion as describe in our previous study [16]. Briefly, a 20% gastric pouch was created around the cardia. Biliopancreatic and roux-limb were 15 and 10 cm respectively. After RYGB reconstruction, the Peterson’s space mesenteric defect **(**Fig. 1**)** in group B, C, D and E were closed using 4–0 non-absorbable suture (Ethicon Prolene Polypropylene Suture), biological glue, 4–0 non-absorbable suture (Polyester suture) and 4–0 absorbable suture (Covidien Polysorb Braided Absorbable Suture) respectively. Closure of mesenteric defect was performed with continuous suturing. In the glue group, we took extreme care to avoid spillage during the application of the glue. The intestine and surrounding tissue were pull away to only release after the glue was completely dried. The Peterson’s space of the group A was left unclosed as control group. Ceftriaxone (75 mg/kg) was injected intraperitoneally as antimicrobial prophylaxis before closing the abdomen. The abdomen was closed in two layer using a 3–0 non-absorbable suture. Operation time was 30 ± 10 min for each rat in all groups. Rats were placed on a heating pad following surgery awaiting recovery from anesthesia. All rats were allowed free access to ad libitum with normal chow and tap water beginning 24 h after surgery.

All rats underwent a laparotomy 8 weeks after surgery to evaluate the adhesion of the mesentery defect and the changes in suture materials. The Peterson’s space mesenteric in all rats in each group were visually inspected to ascertain the degree of adhesion. The degree of adhesion is determined by a semi-quantitative grading method combine Blauer and Linsky’s [17, 18] adhesion scoring system: 0 = no adhesion; 1 = thin adhesion band, easy to separate, or adhesion on the suture plane ≤50%; 2 = thick adhesion band and adhesion on the suture plane >50%; 3 = complete adhesion on the suture plane and difficult to separate; 4 = more than 1 area of thick adhesion zone, adhesion between intestine and/or abdominal wall.

### Statistics

The changes in Food intake, Body weight, and Adhesion scores are expressed as mean ± standard deviation (SD). Differences between the groups were assessed by one-way analysis of variance (ANOVA, LSD post-test). *P*<0.05 was assumed significant difference. Statistics were performed using SPSS, version 18.0, statistical software (SPSS Inc., Chicago, IL).

## Results

### Operative results

The RYGB model was created in all rats in each group. There were no complications during the surgery. All animal survive the surgery and follow-up for 8 weeks.

### Food intake and body weight

**Fig. 2:**
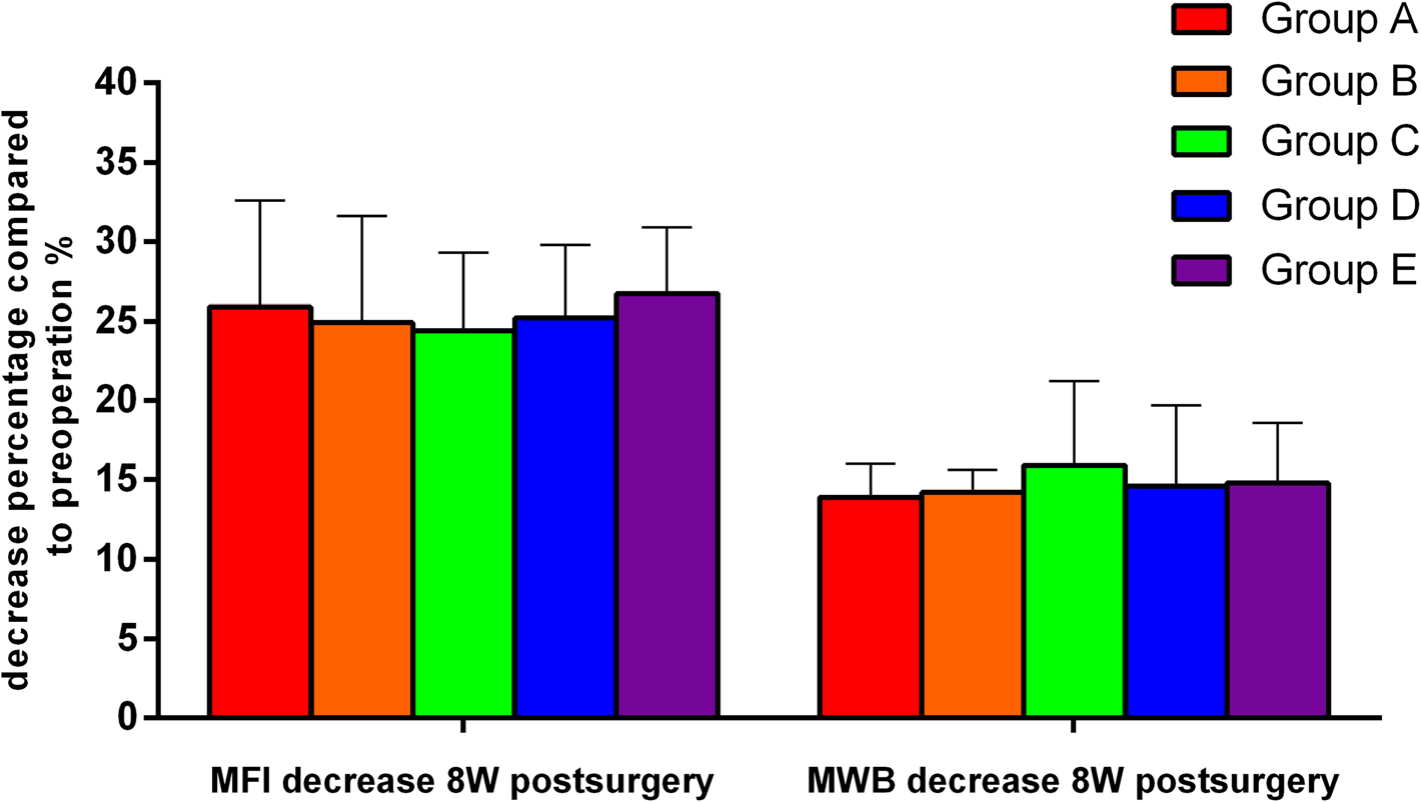
Food intake and body weight decrease (%) at 8 weeks after surgery. The data showed no significant difference was found in the decreased mean food intake (MFI) and mean body weight (MBW) among all groups. Group A (Control group), Group B (non-absorbable Ethicon Prolene Polypropylene Suture), Group C (biological glue, Compon/kangpaite biological adhesive, Beijing), Group D (non-absorbable Ethicon Polyester Suture), Group E (absorbable Covidien Polysorb Braided Absorbable Suture)

Preoperatively, the mean Food intake (g) were; 34.2 ± 2.6, 33.4 ± 3.0, 35.3 ± 3.2, 34.5 ± 2.4, 34.3 ± 2.1 in group A, B, C D and E respectively. The mean perioperative Bodyweight (g) were; 361.7 ± 6.1, 363.6 ± 6.9, 364.4 ± 9.7, 366.0 ± 7.2 and 367.0 ± 9.2 in group A, B, C D and E respectively. Postoperatively, Food intake and body weight decrease significantly in all surgical groups. Food intake decline by 25.9% ± 6.7, 24.9% ± 6.3, 24.4% ± 4.9, 25.2% ± 4.6 and 26.7% ± 4.2% in Group A, B, C, D and E respectively. Decrease in bodyweight were 13.9% ± 2.1, 14.2% ± 1.4, 15.9% ± 5.3, 14.6% ± 5.1 and 14.8% ± 3.8%, in Group A, B, C, D and E respectively at 8 weeks after surgery. The decreased food intake and body weight after surgery did not differ significantly between the groups. **(**Table 1**,** Fig. 2**).**

**Table 1:** Mean food intake and mean bodyweight decreased at 8 week postsurgery compared to presurgery

	Group A	Group B	Group C	Group D	Group E	*P* value
MFI presurgery (g)	34.2 ± 2.6	33.4 ± 3.0	35.3 ± 3.2	34.5 ± 2.4	34.3 ± 2.1	
MFI 8 W postsurgery (g)	25.4 ± 3.7	25.0 ± 2.0	26.6 ± 1.7	25.7 ± 1.2	25.1 ± 1.9	
MFI decrease %	25.9 ± 6.7	24.9 ± 6.3	24.4 ± 4.9	25.2 ± 4.6	26.7 ± 4.2	>0.05
MBW presurgery (g)	361.7 ± 6.1	363.6 ± 6.9	364.4 ± 9.7	366.0 ± 7.2	367.0 ± 9.2	
MBW 8 W postsurgery (g)	311.3 ± 8.0	312.2 ± 9.9	306.7 ± 22.2	312.3 ± 14.6	312.5 ± 12.9	
MBW decrease %	13.9 ± 2.1	14.2 ± 1.4	15.9 ± 5.3	14.6 ± 5.1	14.8 ± 3.8	>0.05

*MFI* Mean food intake, *MBW* Mean bodyweight, *W* Week

### IH and adhesion of the mesenteric defects

**Fig. 3:**
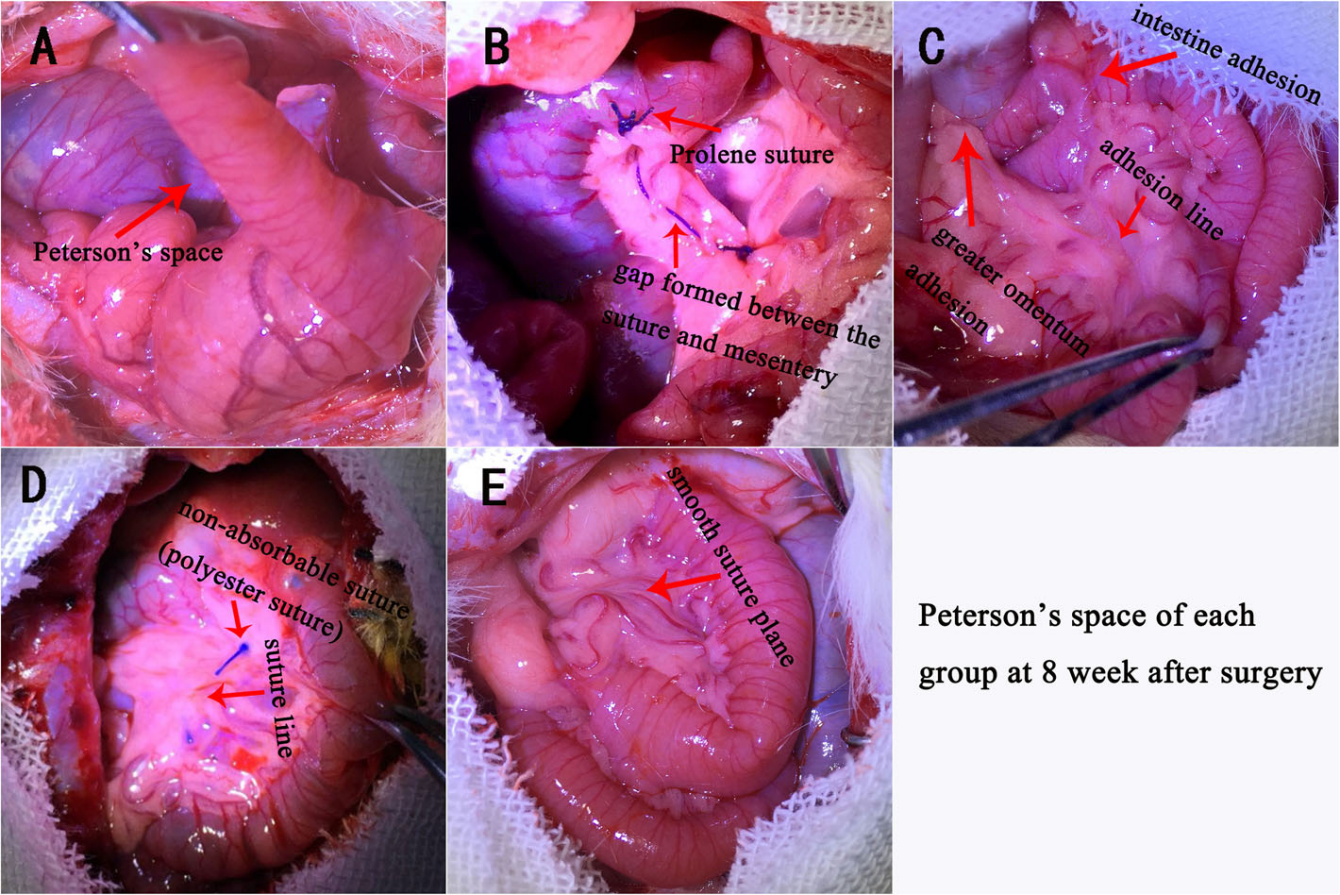
The results of mesenteric defect (Peterson’s space) of each group at 8 weeks after surgery. No IH was found in any group. **a** Control group. The Peterson’s space remains completely visible without any closure or adhesion. **b** Prolene suture group. Multiple gaps were found between prolene suture and the mesentery along the suture line. The gaps range from 0.5 mm to 2 mm. The suture material was visibly present with little adhesion to the mesentery. **c** Glue group. The Peterson’s space was complete closure and multiple adhesions of the small intestine and the greater omentum throughout the area of the glue application. **d** Non-absorbable suture group (Polyester suture). The Peterson’s space had closed completely. The suture was still present, and adhesions along the suture plane was found. **e** The Peterson’s space was completely closed and the suture had completely absorbed leaving a smooth plane along the line of suture. The adhesions between mesentery near sutures were tight

All rats underwent a laparotomy 8 weeks after surgery. No IH was found in any rat and all anastomosis were intact in all rats in each groups. The Peterson’s space remained completely visible without any closure or adhesion at 8 weeks after surgery in Group A (control group). Even though the defect remained completely visible, we did not find any internal herniation. We also did not find any bowel adhesion or obstruction (Fig. 3a)**.** In group B (non-absorbable prolene suture), we found multiple gaps formed between the suture and mesentery along the suture line. The gaps range from 0.5 mm to 2 mm. The suture material was also visibly present with little adhesion to the mesentery in some places along its course. We also did not find any adhesion of the small intestine or adjacent structures along the suture line **(**Fig. 3b). In Group C (Glue) the Peterson’s space was completely closed. However, there were multiple adhesions of the small intestine and the greater omentum throughout the area of the glue application. Nevertheless, there were no visible internal herniation or bowel obstruction **(**Fig. 3c). In Group D (non-absorbable polyester suture) the Peterson’s space mesenteric defect was completely closed. However, the suture was still present. Additionally, there were multiple adhesions along the suture plane **(**Fig. 3d). In Group E (absorbable suture) we also found that the mesenteric defect was completely closed, and the suture was completely absorbed leaving a smooth plane along the line of suture. The adhesions between mesenteries were tight **(**Fig. 3e).

**Fig. 4:**
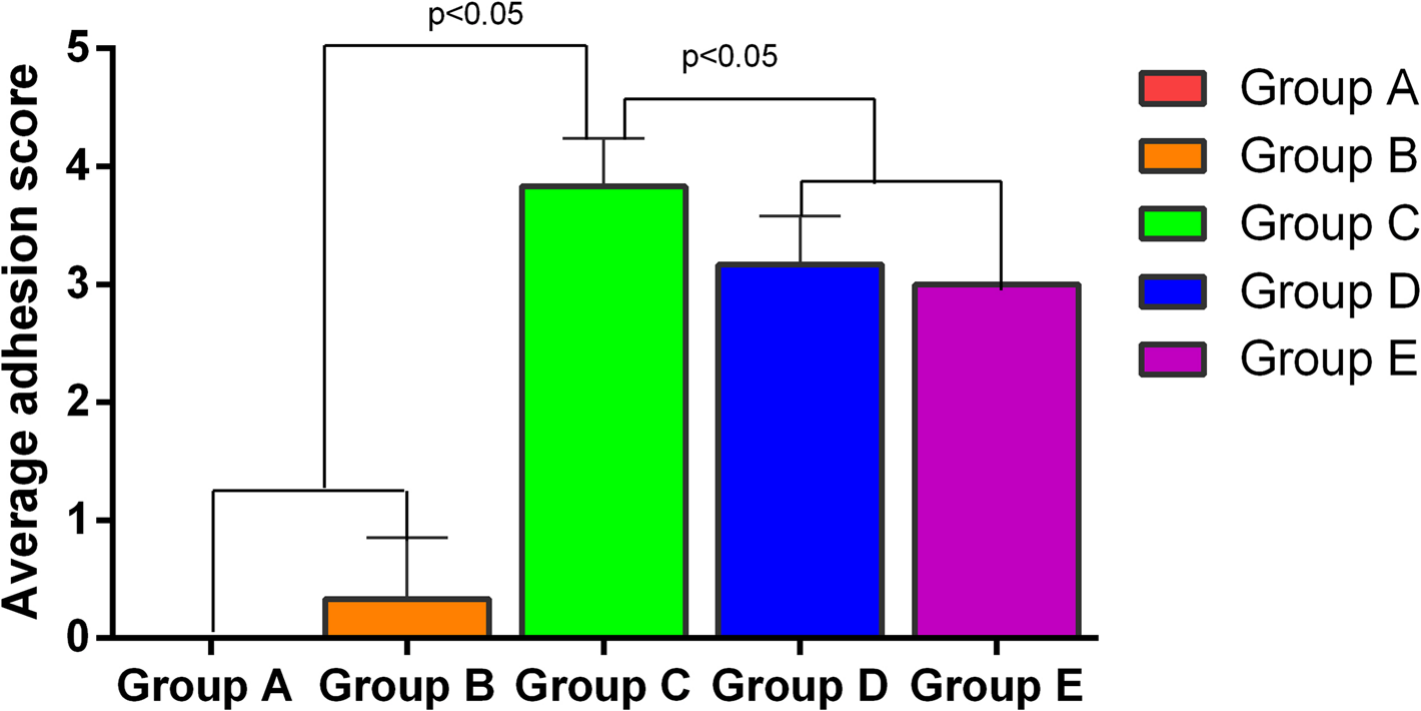
Average adhesion score of each group. Average adhesion scores of Group A and B were 0 and 0.33 ± 0.52 respectively (*p*>0.05). The group C showed the higher adhesion score of 3.83 ± 0.41compared to the other groups (*p*<0.05). Group D and E had similar adhesion scores of 3.17 ± 0.41 and 3.00 ± 0.00 respectively (*p*>0.05). Group A (Control group), Group B (non-absorbable Ethicon Prolene Polypropylene Suture), Group C (biological glue, Compon/kangpaite biological adhesive, Beijing), Group D (non-absorbable Ethicon Polyester Suture), Group E (absorbable Covidien Polysorb Braided Absorbable Suture)

Group C (Glue) showed the highest average adhesion score (3.83 ± 0.41) among all groups. Group A had no adhesion and adhesion score was all 0. The adhesion score in group B was Similar to Group A (<1.0). Group D and E had similar average adhesion score, (3.17 ± 0.41 and 3.00 ± 0.00 respectively, *p*>0.05), but both were significantly lower than group C (*p*<0.05) **(**Fig. 4**)**.

## Discussion

In this study, our results showed that absorbable suture creates a safe adhesion score between the mesentery which is not inferior to non-absorbable sutures.

In our experiment, we found that the mesenteric defects were completely closed in the absorbable and non-absorbable (Group D non-absorbable polyester suture) sutures groups. In the absorbable suture group, a complete absorption of the suture which leaves a smooth plane along the suture may be added advantage. Therefore, we believed that absorbable sutures are safe and not inferior to non-absorbable suture to close mesenteric defect.

Many materials are currently being used by surgeons to close the mesenteric defects. These include various non-absorbable sutures, stapler, biological glue, hernia mesh, hernia clips and so on [4, 9, 11, 19–21]. Some surgeons are accustomed to closing the mesenteric defects using prolene sutures because of its non-absorbability and smoothness. Remarkably, our experiment results indicated that adhesion in prolene group was minimum. Importantly, we found gaps formed between the suture and mesentery along the suture line likely indicating that using prolene sutures may not be safe to close the mesenteric defect probably because of the light tissue response and little adhesion. Moreover, after bariatric surgery this situation is likely exacerbated by the weight loss and the decrease in mesentery fat that occurs.

This phenomenon with Prolene sutures may therefore increase the risk for postoperative internal hernia formation particularly in obesity surgery following the decrease of mesenteric fat as a result of weight loss after gastric bypass. Using another non-absorbable suture (4–0 Polyester suture) we found a complete closure and adhesion of the mesenteric defect although the suture was still present. We want to emphasize that IH may still occurred after operation irrespective of the suture material due to improper suture technique.

Biological glue can also be used to close mesenteric defect based on findings in this study. However, the use of glue was associated with surrounding tissue and intestinal adhesion in the area the glue was applied. The average adhesion score in the glue group was the highest among all groups. Therefore, it is feasible to use the glue to close the mesenteric defect, but the risk of intestinal and surrounding tissue adhesion is high, likely because it is difficult to control the amount of glue during application. But, in the study by Mark Magdy et al., [20] they closed the Petersen’s space mesentery defect using bioabsorbable mesh with fibrin glue fixation with a good result.

It is currently unknown whether closing the mesenteric defect with absorbable sutures creates a safe adhesion compared to non-absorbable suture. The purpose of this study was to try to explore this question. The results of our study showed that Absorbable sutures creates a safe adhesion score between the mesentery which is not inferior to non-absorbable sutures. Additionally, the use of absorbable suture showed a complete closure of the mesentery defect with the sutures completely absorbed leaving a smooth plane along the sutured line. There were no visible bowel adhesions or internal herniation. This indicates that absorbable sutures are safe in closing mesenteric defects.

Gumbs et al. [22] analyzed 152 patients in whom laparoscopic Roux-en-y gastric bypass (LRYGB) was performed. They recorded that jejunojejunal anastomotic obstruction occurred in 7 patients due to small intestine adhesion, which was attributed to the Dacron suture. Their study therefore indicated that non absorbable suture is not a good selection to close the jejunojejunal mesenteric leaves defect.

No matter what suture is used, the closure of the mesenteric defect ultimately depends on the adhesion between mesentery. Comparing all methods used in our experiment, applying absorbable suture and non-absorbable suture (polyester suture) to close the mesenteric defect were equally safe. However, the absorbable suture may be superior to the non-absorbable suture and glue to close the mesenteric defect, because it did not cause extra adhesions perhaps due to the complete absorption.

Here, we need to emphasize that different absorbable sutures require different time for absorption. The time require for absorption of the absorbable suture used in our experiment is about 2 months. So it is unknown if other absorbable sutures (shorter or longer absorbable time) could create safe adhesion in the mesenteric defect. We think a suture with too short absorbable time (one or 2 weeks) may not be suitable for closing the mesenteric defects, because adhesions between mesentery may not have formed or not be firm enough after such a short period of time.

### Limitations

The obvious shortcoming of this study is the use of animal model to perform the experiment which cannot completely represent humans, and absence of internal hernia in any group may be due to the small number of rats. Therefore much larger studies are need.

Although we have used absorbable sutures to close the mesenteric defect in dozens of patients with radical gastrectomy and no IH was found after more than 1 year of follow-up, larger clinical trial or multi-center studies are needed to clarify the safety of absorbable sutures in closing the mesenteric defects.

## Conclusion

We have demonstrated that the application of absorbable sutures to close mesenteric defect creates a safe adhesion score between the mesentery which is not inferior to non-absorbable sutures. Complete absorption which leaves a smooth plane along the suture may be added advantage of absorbable suture based on findings in our experimental study. Therefore, we believed that absorbable sutures are safe to close mesenteric defect.

## Data Availability

The datasets used and/or analysed during the current study are available from the corresponding author on reasonable request.

## Abbreviations

ASMBS: American Society for Metabolic and Bariatric Surgery
IH: Internal hernia
LRYGB: Laparoscopic Roux-en-y gastric bypass
RYGB: Roux-en Y Gastric Bypass
SBO: Small bowel obstruction
